# Prevalent HBV point mutations and mutation combinations at BCP/preC region and their association with liver disease progression

**DOI:** 10.1186/1471-2334-10-271

**Published:** 2010-09-16

**Authors:** Dake Zhang, Sufang Ma, Xin Zhang, Hanqing Zhao, Huiguo Ding, Changqing Zeng

**Affiliations:** 1 Beijing Institute of Genomics, Key Laboratory of Genome Sciences and Information, Chinese Academy of Sciences, Beijing 100029, China; 2 Beijing You'an Hospital, Capital Medical University, Beijing 100069, China; 3 Jinxiang County People's Hospital, Shandong 272200, China; 4 Graduate University of the Chinese Academy of Sciences, Beijing 100049, China

## Abstract

**Background:**

Mutations in the basic core promoter (BCP) and its adjacent precore (preC) region in HBV genome are common in chronic hepatitis B patients. However, the patterns of mutation combinations in these two regions during chronic infection are less understood. This study focused on single base mutations in BCP and preC region and the multi-mutation patterns observed in chronic HBV infection patients.

**Methods:**

Total 192 blood samples of chronic HBV infection patients were included. Direct PCR sequencing on the target region of HBV genome was successfully conducted in 157 samples. The rest 35 samples were analyzed by clone sequencing. Only the nucleotide substitutions with their frequencies no less than 10% were included in multi-mutation analysis with the exception for the polymorphic sites between genotypes B and C.

**Results:**

Five high frequency mutations (≥10%) were found in BCP and preC region. Thirteen types of multi-mutations in one fragment were observed, among which 3 types were common combinations (≥5%). The top three multi-mutations were A1762T/G1764A (36%), A1762T/G1764A/G1896A (11%) and T1753(A/C)/A1762T/G1764A/G1896A (8%). Patients with multi-mutations in viral genomes (≥3) were more likely to have liver cirrhosis or hepatocellular carcinoma (OR = 3.1, 95% CI: 1.6-6.0, P = 0.001). G1896A mutation seemed to be involved in liver disease progression independent of the patient age (OR = 3.6, 95% CI: 1.5-8.6; P = 0.004). In addition, patients with more viral mutations detected (≥3) were more likely to be HBeAg negative (OR = 2.7, 95% CI: 1.1-6.4; P = 0.027). Moreover, G1776A mutation was shown to contribute to HBeAg negativity in our study (OR = 8.6, 95% CI: 1.2-44.9; P = 0.01).

**Conclusions:**

Patients with advanced liver diseases and with HBeAg negativity more likely have multi-mutations in HBV genomes but with different mutation combination patterns. G1896A mutation appears to be independent of infection history.

## Background

The basic core promoter (BCP, nt 1742-1849) and its adjacent precore (preC) region are crucial for replication of HBV. BCP binds various liver factors and preC forms ε structure in pregenomic RNA (pgRNA) as the encapsidation signal [1-3]. Changes in viral replication may influence the progression of liver diseases, particularly in fulminant hepatitis and acute exacerbation of chronic hepatitis [4,5]. Mounting evidence has emerged to demonstrate that BCP and preC mutants are predisposed to severe and progressive liver diseases after HBV infection, causing an increased risk for hepatocellular carcinoma (HCC) [6-10]. For instance, mutations T1762/A1764 and A1899 have been reported to be independent risk factors for HCC [11], and T1653 and/or V1753 mutations are believed to promote the process of liver degradation [12]. However, the association of these mutations with severe symptoms is manifested in certain populations but not in others [13,14].

Studies have been shown that G1896A is involved in HBeAg negativity by introducing a stop codon in the preC region [15]. Although the 1762T/1764A double mutation, commonly occurring in HBeAg-negative patients, was observed *in vivo *to suppress the production of preC mRNA independent of G1896A, recent *in vitro *research suggested other single site substitutions rather than these two may be responsible for the reduction of HBeAg expression [5,16,17]. Unknown mutations in this core promoter may impede the seroconversion of HBeAg during antiviral treatment [18].

In the BCP and preC regions, multi-substitutions further complicate mutation research. Triple core promoter mutations C1753T/A1762T/G1764A occurred more commonly in genotype C compared with genotype B [19]. For genotype D, A1757 mutants were prone to accompany with the T1764/G1766 double mutation [20]. *In vitro *experiments have shown multi-mutations may increase viral replication efficiency in Lamivudine resistant strains [21]. However, the mutation combination in BCP and preC and its clinical significance are less understood in chronic HBV infection patients.

This study focused on substitutions in BCP and preC regions and their combinations in different stages of chronic HBV related liver diseases.

## Methods

### Patients and blood samples

A total of 192 chronic HBV infection patients were enrolled at You'an Hospital (Beijing, China) and Jinxiang County People's Hospital (Shandong Province, China) (Additional file 1, Table S1). A diagnostic workup was performed including physical examination, laboratory and or liver pathology according to the criteria suggested by Chinese Medical Association for Liver Diseases in 2005[22]. Liver function test and serum HBV marker screening were conventionally conducted. No patient had co-infection with hepatitis C virus, hepatitis D virus, or human immunodeficiency virus. Basic patient characters have been summarized in Table 1. Blood samples (5 ml each) were collected, and cells and sera were then separated and stored at -20°C. The study was approved by the Ethics Committees of the institutions, and informed consent was obtained from all patients.

**Table 1:** Clinical characteristics and viral mutations of patients.

**Group**			**Advanced liver diseases**			**Total^Δ^**	***p*-value**
				
**Diagnosis**	**ASC**	**CH**			**HCC**		
				
Patient (No.)	13	75	14	44	11	157	-
Gender (Male/Female)	4/9	56/18	12/2	34/10	10/1	116/40	**0.006**^b^
Age (years, Mean ± SD)	28 ± 9	35 ± 12	37 ± 9	47 ± 11	51 ± 6	38 ± 13	< 0.0001*
HBV DNA titer (lg, Mean ± SD)	6 ± 2	7 ± 2	7 ± 1	7 ± 1	6 ± 1	7 ± 1	0.317*
ALT (U/L, Mean ± SD)	26 ± 24	255 ± 343	212 ± 199	107 ± 127	123 ± 151	184 ± 273	0.006*
AST (U/L, Mean ± SD)	33 ± 16	124 ± 187	136 ± 181	99 ± 104	162 ± 156	114 ± 161	0.237*
nt 1753 (m/wt)	0/13	8/67	2/12	12/32	3/8	25/132	0.053^b^
nt 1762 (m/wt)	1/12	51/24	14/0	32/12	10/1	108/49	**< 0.0001**^b^
nt 1764 (m/wt)	1/12	51/24	13/1	38/6	9/2	112/45	**< 0.0001**^b^
nt 1776 (m/wt)	0/13	3/72	0/14	5/39	0/11	8/149	0.214^b^
nt 1803 (m/wt)	0/13	2/73	0/14	6/38	1/10	9/148	0.082^b^
nt 1846 (m/wt)	0/13	9/66	1/13	6/38	2/9	18/139	0.617^b^
nt 1896 (m/wt)	0/13	17/58	7/7	13/31	6/5	43/114	**0.009**^b^
							
Mutations≥3 (Y/N)^@^	0/13	26/49	10/4	24/20	6/5	66/91	**0.001**^b^

ASC, asymptomatic carrier; CH, chronic hepatitis; LF, liver failure; LC, liver cirrhosis; HCC, hepatocellular carcinoma; SD, standard deviation; **Δ**, samples with missing values were denoted in additional file 1, Table S1; b, chi-square test; *, One way ANOVA, ^@^, only included sites with minor allele frequency greater than 5%.

### Serological HBV marker detection

Serological markers were detected by electrochemiluminescence immunoassay on a Roche E170 modular immunoassay analyzer following the manufacturer's protocols (Roche Diagnostics, Germany).

### HBV DNA quantification

Real time PCR was performed to determine viral DNA titers using an FQ-PCR Kit for HBV (DaAn Gene Co., China) performed in a GeneAmp 5700 Sequence Detection System (PE Applied Biosystems, USA).

### Viral DNA extraction

Viral DNA was extracted from 200 μL sera per sample using an AccuPrep Genomic DNA Extraction Kit (Bioneer, Korea) or QIAamp MinElute Virus Spin (Qiagen, Germany) as instructed in the manufacturer's manuals. All DNA samples were stored at -20°C before thawing for PCR.

### Fragment amplification

All PCRs were performed in 20 μL or 50 μL reaction mixtures containing specimen DNA. PCR for amplification of target regions was performed with a hot start at 95°C for 150 s, followed by 35 cycles of denaturation at 94°C for 1 min, annealing at 58°C for 90 s, and elongation at 72°C for 3 min. All reactions were performed on a PTC-200 Peltier Thermal Cycler (MJ Research, USA). The primers employed were CP5: 5'-CTTCGTCTGCGAGGCGAGGG-3' (nt 2381-2400) and CP22: 5'-GAGACCACCGTGAACGCCCA-3' (nt 1611-1630).

### PCR and clone sequencing

PCR products were purified with a Montage PCR96 column (Millipore, USA). The final DNA concentration in the sequencing reaction was 10 ng/μL. DNA sequencing was carried out on a Prism 3730 (ABI, USA). Contigs were assembled using SeqMan (DNASTAR, USA) and all the sequences were aligned by ClustalW for further analysis (all sequences analyzed in this paper have been submitted to GenBank) (Additional file 1, Table S3).

The PCR products were first purified with a Takara gel purification kit and were then ligated into pMD18 T vector (Takara Bio, Japan). Vectors were subsequently transfected into DH5α cells and white/blue colony selection was used to detect recombinant vectors. Inserts from positive clones were PCR amplified with primers RV-M/M13-47 according to the manufacturer's instructions to verify the target fragments.

### Viral genotyping

The phylogenetic tree (Additional file 1, Figure S1) was built for all 157 sequences and 858 strains from 8 genotypes retrieved from the NCBI database, using Mega 4 [23] and annotated by TreeDyn [24]. All the fragments of BCP and preC were from whole genomes in the NCBI database with genotypes annotated.

### Statistical Analysis

Statistical analysis was performed using SPSS software (version 13.0; SPSS Inc, USA). Logistic regression was used for evaluating the roles of candidate clinical factors and viral mutations in liver disease progression and HBeAg negativity.

## Results

### High prevalent mutations in BCP and preC regions

**Figure 1:**
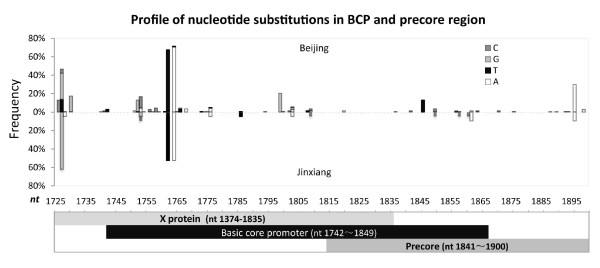
**Nucleotide substitution profiles in BCP and preC**. The top panel illustrates frequencies of all mutations in the BCP and preC of viral genomes. Hot spots of mutations are largely congruent in patients from Beijing (upper half) and Jinxiang (lower half) except for different mutation rates at certain positions. The bottom panel demonstrates the positions of the X gene, BCP and preC gene in the region. The overlapped part for all three regions is unlikely to mutate.

A total of 300 viral fragments from nt 1725 to 1900, covering the BCP and preC regions in the HBV genome, were analyzed. As shown in Table 1, direct sequencing resulted 157 fragments from patients of 13 asymptomatic carrier (ASC), 75 chronic hepatitis (CH), 44 liver failure (LF), and 11 hepatocellular carcinoma (HCC). LF, LC, and HCC were defined as advanced liver diseases (ALD). The rest 143 sequences came from clone sequencing in which 95 clones were from 17 samples of LC patients and 48 from 18 samples of HCC patients. Figure 1 illustrates the nucleotide substitution patterns identified by PCR sequencing. Similar mutation profiles were shown in samples collected in Beijing (136) and in a relatively isolated town 600 km south from Beijing (21). Mutations were rarely seen in gene overlapping regions. About 82% (128/157) sequences were genotype C and the rest were type B by clustering with reported sequences in NCBI database (Additional file 1, Figure S1). There were 12 nucleotide substitutions with their mutation rates over 5% (Additional file 1, Table S2). Further comparison of these sites with 233 genotype B and 311 genotype C of HBV sequences from NCBI database demonstrated that five were at the genotype specific positions and were therefore precluded from further analysis (Table 2). Interestingly, in three previously reported common mutations G1764A, A1762T, and G1896A in this region, the first two types were observed with very high prevalence in these samples (70% and 67%, respectively) (Additional file 1, Table S2).

**Table 2:** Genotype specific nucleotide positions.

*nt*	genotye B (NCBI)				genotye C (NCBI)			
		
	A	T	G	C	A	T	G	C
1726	32%	0%	0%	**68%**	**88%**	0%	0%	13%
1727	3%	**91%**	4%	2%	**63%**	15%	22%	0%
1730	2%	0%	**95%**	3%	2%	0%	15%	**83%**
1752	**54%**	3%	43%	0%	**90%**	2%	7%	1%
1799	0%	1%	**61%**	38%	0%	1%	9%	**90%**

### G1896A mutation in liver disease progression

Next we analyzed all substitutions in the ALD group to reveal possible risk factors for liver disease progression. In univariate binary logistic regression analysis, all the top five high occurrence mutations seemed to relate to ALD, including T1753A/C (OR = 3.2, 95% CI: 1.3-7.9, P = 0.013), A1762T (OR = 3.1, 95% CI: 1.5-6.5, P = 0.003), G1764A (OR = 4.8, 95% CI: 2.1-10.9, P < 0.001), T1803A/G (OR = 5.1, 95% CI: 1-25.4, P = 0.058), and G1896A (OR = 2.4, 95% CI: 1.2-5.0, P = 0.015). However, in patients older than 35, age appears to be a significant factor in disease progression (OR = 5.5, 95% CI: 2.6-11.5, P < 0.0001), raising the question if the significance of above mutations in ALD was simply due to the accumulation of mutations during long infection history. Indeed subsequent multivariate binary logistic regression analysis indicated that only the mutation G1896A significantly correlated to the disease progression independent of age (P = 0.007, Table 3). Patients with the G1896A (mean age 40 ± 11) had similar average age with those without this mutation (mean age 38 ± 14) but more had ALD (FET, P = 0.005, Table 1).

**Table 3:** Clinical status and viral mutations in patients of HBeAg negative and positive.

Factors	HBeAg^- ^(n = 25)	HBeAg^+ ^(n = 131)	Total^Δ^(n = 156)	*p-*value
Gender (Male/Female)	21/4	92/38	113/42	0.13^b^
Age (years, Mean ± SD)	42 ± 14	38 ± 13	39 ± 14	0.165*
Clinical status (ALD/PLD)	16/9	50/81	66/90	**0.015^b^**
HBV DNA titer (lg, Mean ± SD)	6 ± 1	7 ± 2	7 ± 1	0.127*
ALT (U/L, Mean ± SD)	258 ± 466	178 ± 230	191 ± 279	0.199*
AST (U/L, Mean ± SD)	203 ± 328	104 ± 111	119 ± 165	0.008*
nt 1753 (m/wt)	5/20	18/113	23/133	0.296^b^
nt 1762 (m/wt)	19/6	85/46	104/52	0.2^b^
nt 1764 (m/wt)	19/6	89/42	108/48	0.292^b^
nt 1776 (m/wt)	5/20	3/128	8/148	**0.003^b^**
nt 1846 (m/wt)	6/19	10/121	16/140	**0.024^b^**
nt 1896 (m/wt)	12/13	28/103	40/116	**0.007^b^**
nt 1899 (m/wt)	2/23	2/129	4/152	0.121^b^
				
Mutation≥3 (Y/N)	15/10	47/84	62/94	**0.022^b^**

ALD, advanced liver diseases; PLD, Preliminary liver diseases; SD, standard deviation; Δ, samples with missing values in additional file 1, Table S1; b, chi-square test; *, Fisher exact test; Y, number of mutations is at least 3; N, number of mutations is less than 3.

### G1776A statistically correlated to HBeAg negativity

Although having their viral DNA load detected (> 10^3 ^copies/ml), some patients in our study were shown to be HBeAg negative, suggesting their HBeAg negativity was not caused by the disappearance or reduction of viral replication. To identify possible mutations correlating to HBeAg negativity, clinical status and viral substitution patterns in BCP and preC were compared between HBeAg negative and positive groups. As shown in Table 4, HBeAg negative patients contained more mutations in viral genomes and were also prone to develop more severe liver diseases such as liver cirrhosis and carcinoma. Additionally, these patients seemed also to have higher levels of aspartate transaminase (AST) (chi-square test, P = 0.003).

**Table 4:** HBV mutations responsible for HBeAg negativity by multivariate logistic regression.

Step	Factors	B	**S.E**.	Wald	df	P-value	OR	95% CI for OR	
								
								Lower	Upper
1^a^	nt 1776 (m)	2.5	0.8	9.6	1	**0.002**	12.4	2.5	60.6
	nt 1846 (m)	1.0	0.6	2.4	1	0.118	2.7	0.8	9.4
	nt 1896 (m)	1.1	0.6	3.9	1	**0.049**	3.0	1.0	8.8
									
	Mutation≥3 (Y)	0.2	0.6	0.1	1	0.727	1.2	0.4	3.7
									
	Constant	-2.3	1.0	5.6	1	0.018	0.1		

1^a^, denotes Variable(s) entered in step 1; B, regression coefficient; S.E., standard error; OR, odds ratio; CI, confidence interval; m, mutant; wt, wild type; Y, number of mutations is at least 3; N, number of mutations is less than 3.

We then did regression analysis for all common mutations (≥5%). Univariate binary logistic regression analysis showed significant correlation of several substitutions with the HBeAg negativity, including G1776A (OR = 8.1, 95% CI: 1.7-39; P = 0.009), A1846T (OR = 3.8, 95% CI: 1.2-11.8; P = 0.02), G1896A (OR = 3.5, 95% CI: 1.4-8.6; P = 0.007), and the number of individual point mutations ≥ 3 (OR = 2.8, 95% CI: 1.1-6.8; P = 0.027). By contribution of G1776A to HBeAg negativity was further verified in multivariate binary logistic regression analysis G1896A, a common mutation known to introduce a stop codon in e antigen, showed tendency in correlation to HBeAg negativity (P = 0.055; Table 5). Moreover, within a recently identified binding site for the transcription factor FXRalpha [25], G1776A was verified to associate with the HBeAg negativity significantly (P = 0.010). Therefore, nt 1776 appears to be a novel candidate corresponding to the loss of e-antigen.

**Table 5:** HBV mutations responsible for HBeAg negativity by multivariate logistic regression.

Step	Factors	B	**S.E**.	Wald	df	P-value	OR	95% CI for OR	
								
								Lower	Upper
1^a^	nt 1776 (m)	2.2	0.8	6.6	1	**0.010**	8.6	1.7	44.9
	nt 1846 (m)	1.0	0.6	2.4	1	0.121	2.7	0.8	9.1
	nt 1896 (m)	1.1	0.6	3.9	1	**0.055**	3.0	1.0	8.8
									
	Mutation≥3 (Y)	0.2	0.6	0.1	1	0.662	1.2	0.4	3.7

1^a^, denotes Variable(s) entered in step 1; B, regression coefficient; S.E., standard error; OR, odds ratio; CI, confidence interval; m, mutant; wt, wild type; Y, number of mutations is at least 3; N, number of mutations is less than 3.

### Common multi-mutations in BCP and preC regions

**Figure 2:**
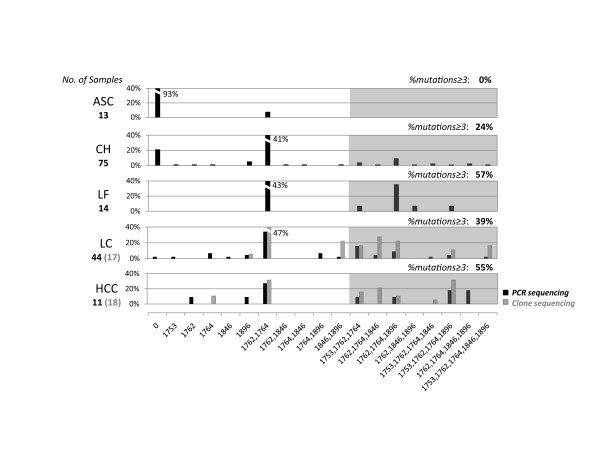
**Combination patterns of viral mutations in patients with different diagnoses**. Point utations identified in the PCR and clone sequencing were demonstrated along the x-axis. "0" represents strains without mutations in indicated sites. Only certain combinations were detected among a number of calculated possibilities. Grey parts illustrated combinations of no less than 3 sites, and their frequencies were on the right top. Mutation profiles were more complicated in patients of liver failure (LF), liver cirrhosis (LC) and hepatocellular carcinoma (HCC) than those of asymptomatic carrier (ASC) and chronic hepatitis (CH). Mutation combinations in LC and HCC patients were identified by both direct PCR sequencing and clone sequencing.

Next we analyzed the effect of multi-mutations in target region. First we defined multi-mutations detected in a single fragment as the mutation combination or the combination in short. Five point mutations (1753, 1762, 1764, 1846, and 1896) with their rates larger than 10% were included in combination analysis. Nineteen categories containing wild type, single base mutations and observed combinations were resulted as illustrated in Figure 2. The top three combinations in all 192 patients were the double mutation A1762T/G1764A (36%), the triple mutation A1762T/G1764A/G1896A (11%), and the quadruple mutation T1753(A/C)/A1762T/G1764A/G1896A (8%).

**Figure 3:**
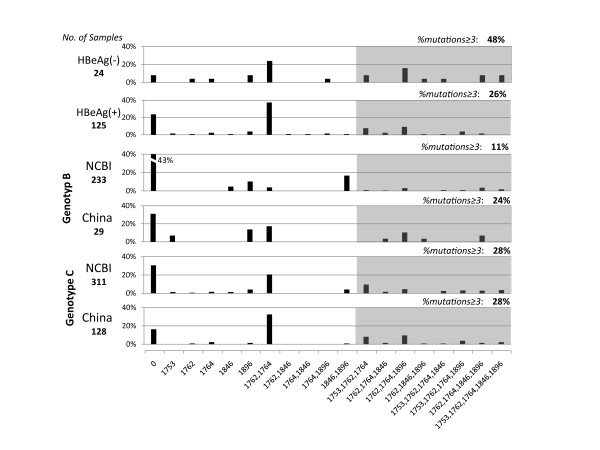
**Combination patterns of viral mutations in HBeAg(+/-) patients and genotype B/C strains**. Point mutations identified in the PCR and clone sequencing were demonstrated along the x-axis. "0" represents strains without mutations in indicated sites. Grey parts illustrated combinations of no less than 3 sites, and their frequencies were on the right top.HBeAg negative(-) patients demonstraed more complicated mutation combinations than those of HBeAg postive(+). Mutation profiles were similar in genotype B and C strains in both NCBI database and samples obtained in this study.

While comparing the results of PCR sequencing and clone sequencing, we noticed an interesting phenomenon. All the mutation combinations observed in PCR sequencing were also detected in single strains obtained by clone sequencing, suggesting that point mutations tended to coexist in single genomes rather than to occur in various fragments among viral quasispecies (Figure 2). In addition, these combinations were also seen in NCBI sequences of both genotype B and C (Figure 3).

### Multi-mutations in ALD patients

As shown in Figure 2, the distribution of above multi-mutations was different between ALD and other patients (ASC and CH) (FET, P < 0.0001). Patients with the combinations of more than 3 mutations were more likely to have ALD (OR = 3.1, 95% CI: 1.6-6.0, P = 0.001), although only one combination (T1753(A/C)/A1762T/G1764A) was dominant in these patients (FET, P = 0.025). Furthermore, ALD patients (mean age 46 ± 11) were older than others (mean age 33 ± 12; T-Test, P < 0.0001) and the patients with no less than 3 viral mutations (mean age 42 ± 12) were also older than the rest (mean age 36 ± 13) (P = 0.009), suggesting that the age, which usually represent the infection history appears to be a correlating factor in liver disease progression.

## Discussion

### Candidate mutations responsible for the loss of e antigen

Previous studies have suggested high mutations in BCP and preC regions are major cause for the loss of HBeAg but without true disappearance or reduction of viral load. Thus far only G1896A, which leads a 2/3 truncated e antigen by a stop codon caused by mutation, was verified to be responsible for HBeAg negativity. In this study, we showed that G1776A is a new candidate mutation correlating to HBeAg negativity. Since nt1776 localizes at the binding site to transcription factor FXRalpha, which promotes the expression of e antigen [25], one possible mechanism for G1776A mutation is to interrupt normal transcription of preC region. As this is the first time proposing possible role of G1776A, further verification is needed in larger sample size.

### Viral mutation accumulation in patients with long history of infection

Evidenced by the appearance of some mutations earlier than others during the infection, the accumulation of mutations in viral genome had been observed in previous studies [26]. Our results suggested these accumulated mutations fell into two categories. Mutations in first type were adaptive to host system after long infection but had little effect in the disease progression. A feature of those mutations, including T1753A/C, G1764A and A1762T, is their significance as risk factors in disease progression only shown by univariate analysis but not in the multivairate model when patient age was considered. The second type, such as G1896A, has high occurrence in ALD patients and is independent from infection history, suggesting mutants carrying this substitution may lead to worsening symptoms once they appear.

The occurrence rates of T1764 and A1762 were very high in our study (70% and 67%, respectively). This is perhaps because the major path of HBV infection is vertical transmission in China and patients in their ages of 30 s are usually already with long infection history [9]. The effects of G1762A and A1764T in disease progression had attracted attentions especially in genotypes B and C [27,28], however our study suggests that the appearance of these mutations may just be a sign of long infection history and may not be very important in disease prognosis.

### Common combinations of prevalent substitutions in BCP and preC

Few studies have been conducted about combination of common nucleotide substitutions, and here we present multi-mutations with high frequencies in patients with various diagnoses. Mutation combination appeared as a common type of variation in HBV genome regardless of genotypes. Despite many possible patterns, only 13 combinations were observed based on 5 common substitutions in our study. These limited multi-mutation patterns indicate only certain combinations may have selective advantage for viral functions which makes it possible to interrogate their effects in disease progression. However, limited samples for each mutation combination reduce the statistic power to dissect their distribution in patients with different diagnoses. Therefore, larger sample size should be considered in further investigation

## Conclusions

Based on 192 patients from northern China, analysis of common mutations and their combinations were conducted in BCP and preC regions. G1896A was indicated to be associated with liver disease progression independent of the infection history. G1776A mutation was statistically responsible for HBeAg negativity. Mutation profiles of viral genomes were complicated in ALD patients and HBeAg negative patients. Common mutation combinations were observed by both PCR and clone sequencing, indicating their coexistences in single fragment.

## Competing interests

The authors declare that they have no competing interests.

## Authors' contributions

The original data process and computation, and manuscript preparation were undertaken by DZ. SM, XZ and HZ collected samples and helped with sequencing experiments and data analysis. HD and CZ directed the project and prepared the bulk of the manuscript. All the authors read and approved the final manuscript.

## Pre-publication history

The pre-publication history for this paper can be accessed here:

http://www.biomedcentral.com/1471-2334/10/271/prepub

## Supplementary Material

Additional file 1 **Supplementary figures and tables**. This file contains the following figures and tables: Figure S1. Phylogenetic tree of BCP and preC regions in HBV genome (nt1725-1900). Table S1. Patient information. Table S2. Mutation profile in nt 1725-1900 based on PCR. Table S3. GeneBank accession numbers for HBV sequences submitted in this study. Click here for file
