# Supplementary material for: Prevalent HBV point mutations and mutation combinations at BCP/preC region and their association with liver disease progression
Source: BMC Infect Dis. 2010 Sep 16;10:271. doi: 10.1186/1471-2334-10-271 (PMC2949759; doi:10.1186/1471-2334-10-271)
Supplement: Additional file 1 — Supplementary figures and tables. This file contains the following figures and tables: Figure S1. Phylogenetic tree of BCP and preC regions in HBV genome (nt1725-1900). Table S1. Patient information. Table S2. Mutation profile in nt 1725-1900 based on PCR. Table S3. GeneBank accession numbers for HBV sequences submitted in this study. [file 1471-2334-10-271-S1.DOC]

**Supplementary figures and tables**

**Figure S 1. Phylogenetic tree of BCP and preC regions in HBV genome (nt1725-1900).** Terminal branches of the tree were drawn in gradient gray for 8 genotypes (A-H), among which the fonts with smaller sizes indicate lower proportion of this genotype in the cluster. Branches with gray dots at the tip indicate the 157 sequences analyzed in this study (dots of dark gray show Beijing cases and of light gray as Jinxiang samples, respectively).

**Table S1. Patient information.**

| **Patient** | **mutation summarization** | **Diagnosis** | **Gender** | **AGE** | **logDNA** | **HBeAg** | **ALT** | **AST** | **Geno** |
| --- | --- | --- | --- | --- | --- | --- | --- | --- | --- |
| 3 | 1762,1764,1896 | 4 | 0 | 50 | 7.1 | 1 | 96 | 1.48 | C |
| 8 | 0 | 3 | 1 | 28 | 6.9 | 1 | 25 | 22 | B |
| 14 | 1896 | 3 | 1 | 26 | -1.0 | 1 | 625 | 216 | B |
| 16 | 1762,1764,1846,1896 | 3 | 1 | -1 | 7.9 | 1 | 286 | 104 | C |
| 24 | 1764,1766,1896 | 6 | 1 | 40 | -1.0 | 0 | 22 | 17 | C |
| 33 | 1753,1762,1764,1846 | 3 | 1 | 41 | 7.3 | -1 | 91 | 41 | C |
| 38 | 1896 | 3 | 1 | 29 | 8.9 | 1 | 235 | 172 | B |
| 50 | 1753,1762,1764 | 7 | 1 | 49 | -1.0 | 1 | 106 | 148 | C |
| 69 | 1762,1764 | 3 | 1 | 35 | 8.6 | 0 | 2023 | 1454 | B |
| 77 | 1762,1764 | 6 | 1 | 42 | 4.9 | 0 | 54 | 48 | C |
| 79 | 1762,1764 | 6 | 1 | 49 | -1.0 | 1 | 134 | 76 | C |
| 81 | 1762,1764 | 3 | 1 | 25 | 7.7 | 1 | 39 | 28 | C |
| 83 | 0 | 3 | 1 | 17 | -1.0 | 1 | 18 | 19 | C |
| 84 | 1762,1764 | 3 | 1 | 47 | -1.0 | 1 | 60 | 29 | C |
| 85 | 1762,1764 | 4 | 1 | 58 | 4.9 | 0 | 628 | 701 | C |
| 88 | 1762,1764,1896 | 6 | 0 | 34 | 8.3 | 0 | 83 | 193 | C |
| 95 | 1764 | 3 | 1 | 31 | 5.6 | 1 | 951 | 219 | C |
| 125 | 1803,1846,1896 | 6 | 1 | 29 | 7.8 | -1 | 175 | 114 | C |
| 135 | 1896 | 5 | 0 | 50 | 6.2 | 1 | 155 | 111 | C |
| 143 | 1762,1764,1803 | 3 | 1 | 39 | 7.3 | 1 | 113 | 128 | C |
| 144 | 1753,1762,1764 | 3 | 1 | 57 | 7.5 | 1 | 104 | 133 | C |
| 145 | 1762,1764 | 3 | 0 | -1 | 6.3 | 1 | 61 | 153 | C |
| 152 | 1762,1764,1846 | 3 | 1 | 53 | 7.4 | 1 | 115 | 59 | C |
| 160 | 1753,1762,1764 | 6 | 1 | 46 | -1.0 | 1 | 37 | 27 | C |
| 163 | 1762,1764,1899 | 3 | -1 | -1 | -1.0 | 1 | 29 | 31 | C |
| 172 | 0 | 3 | 1 | -1 | 7.2 | 1 | 84 | 43 | C |
| 182 | 1762,1764 | 3 | 1 | 30 | 6.7 | 1 | 260 | 123 | C |
| 204 | 1762,1764 | 3 | 1 | 26 | -1.0 | -1 | 106 | 53 | C |
| 210 | 1764,1766 | 6 | 1 | 41 | 5.2 | 1 | 207 | 85 | C |
| 213 | 1762,1764,1896 | 4 | 1 | 32 | 6.1 | 1 | 587 | 107 | C |
| 217 | 1762,1764 | 3 | 1 | 33 | 7.0 | 1 | 295 | 100 | C |
| 218 | 1762,1764,1896 | 4 | 1 | 32 | 6.4 | -1 | 95 | 62 | C |
| 221 | 1762,1764 | 7 | 1 | 50 | -1.0 | 1 | 41 | 45 | C |
| 226 | 1766,1896 | 6 | 1 | 36 | 5.9 | 0 | 775 | 553 | C |
| 234 | 1753,1762,1764 | 6 | 1 | 38 | -1.0 | 1 | 59 | 73 | C |
| 239 | 1762,1764 | 3 | 1 | 34 | 6.4 | 1 | 259 | 113 | B |
| 240 | 1762,1764,1896 | 3 | 1 | 25 | 8.2 | 1 | 450 | 95 | B |
| 255 | 1762,1764,1803 | 4 | 1 | 29 | -1.0 | 1 | 60 | 61 | B |
| 256 | 1762,1764,1896 | 7 | 1 | 55 | -1.0 | 1 | 46 | 98 | C |
| 259 | 1762,1764 | 3 | 1 | 32 | 7.4 | 1 | 86 | 44 | C |
| 260 | 1762,1764,1896 | 3 | 0 | 29 | 7.7 | 1 | 165 | 68 | C |
| 261 | 1753,1762,1764,1846,1896 | 3 | 1 | 52 | -1.0 | 0 | 1117 | 486 | C |
| 262 | 1896 | 7 | 1 | 48 | 5.0 | 0 | 37 | 50 | C |
| 272 | 1762,1764,1803,1846,1896 | 7 | 1 | 54 | -1.0 | 0 | 36 | 72 | B |
| 273 | 1762,1764,1803 | 4 | 1 | 35 | 6.9 | 1 | 195 | 133 | B |
| 277 | 1762,1764 | 3 | 1 | 28 | 5.0 | 1 | 842 | 240 | C |
| 278 | 1762,1764 | 6 | 1 | 51 | -1.0 | 1 | 34 | 41 | C |
| 295 | 1762,1764 | 3 | 1 | 26 | -1.0 | 1 | 123 | 52 | C |
| 296 | 1762,1764,1846,1896 | 7 | 1 | 55 | 6.0 | 0 | 138 | 235 | C |
| 297 | 1762,1764,1896 | 4 | 1 | 45 | 8.2 | 1 | 168 | 323 | C |
| 299 | 1727 | 3 | 0 | -1 | 6.9 | 1 | 50.4 | 31.1 | C |
| 306 | 1753,1762,1764,1776,1803 | 5 | 1 | 37 | -1.0 | 0 | -1 | -1 | C |
| 314 | 1762,1764 | 3 | 1 | 44 | -1.0 | -1 | 47 | 41.2 | C |
| 321 | 1762,1764,1846 | 6 | 0 | -1 | 5.2 | 1 | 51 | 48 | C |
| 340 | 1762,1764 | 7 | 0 | 58 | -1.0 | 1 | 430 | 520 | C |
| 342 | 1762,1764,1896 | 4 | 1 | 32 | -1.0 | 1 | -1 | -1 | C |
| 344 | 1753,1762,1764 | 6 | 1 | 58 | -1.0 | 1 | 88.4 | 97.9 | C |
| 345 | 1753,1762,1764 | 3 | 1 | 34 | 3.0 | 1 | 29.6 | 29.3 | C |
| 351 | 1762,1764,1846 | 6 | 1 | 38 | 6.1 | 1 | 42.9 | 60.2 | B |
| 352 | 0 | 3 | 1 | 18 | 6.7 | 1 | 19.4 | 27.6 | B |
| 353 | 0 | 3 | 1 | 38 | 7.2 | 1 | 49.8 | 25.2 | C |
| 356 | 1762,1764,1896 | 3 | 1 | 40 | 6.2 | 1 | 73.9 | 54.7 | C |
| 360 | 1896 | 3 | 0 | 35 | 5.9 | 1 | 98.1 | 61.3 | B |
| 366 | 1762,1764,1776 | 3 | 1 | 24 | 5.5 | 0 | 109.5 | 52 | C |
| 367 | 1762,1764 | 3 | 1 | 36 | 6.3 | 1 | 102.9 | 51 | C |
| 370 | 1753,1762,1764 | 6 | 1 | 40 | 7.1 | 1 | 51.7 | 47.5 | C |
| G005 | 1762,1764 | 6 | 1 | 31 | 8.5 | 1 | 32 | 41 | C |
| G045 | 1753,1762,1764,1846,1899 | 3 | 0 | 85 | 7.8 | 0 | 63 | 49 | C |
| G057 | 1762,1764,1896 | 3 | 1 | 39 | 5.1 | 1 | 1095 | 477 | C |
| G060 | 1762 | 3 | 1 | 68 | 7.5 | 1 | 205 | 100 | C |
| G061 | 1762,1764,1846,1896 | 3 | 1 | 45 | 7.1 | 1 | 628 | 292 | B |
| G067 | 1762,1764 | 3 | 1 | 19 | 8.3 | 1 | 763 | 375 | C |
| G069 | 1762,1764,1896 | 3 | 1 | 19 | 6.9 | 1 | 165 | 150 | C |
| G071 | 1762,1846,1896 | 3 | 0 | 26 | 7.1 | 0 | 154 | 86 | C |
| G072 | 1753,1762,1764,1896 | 4 | 1 | 31 | 7.6 | 1 | 107 | 53 | C |
| G073 | 1762,1764,1896 | 3 | 0 | 33 | 5.6 | 0 | 73 | 42 | B |
| G075 | 0 | 3 | 1 | 44 | 7.9 | 1 | 43 | 29 | C |
| G076 | 1762,1764 | 3 | 1 | -1 | 7.5 | 1 | 60 | 52 | C |
| G077 | 1762,1764 | 3 | 0 | 43 | 8.0 | 1 | 630 | 322 | C |
| G079 | 1762,1764,1896 | 6 | 1 | 45 | 8.2 | 1 | 289 | 92 | C |
| G174 | 1753,1762,1764 | 6 | 1 | -1 | -1.0 | 0 | 29 | 63 | C |
| G175 | 1762,1764 | 6 | 0 | 33 | 6.2 | 1 | 324 | 482 | C |
| G176 | 1762,1764 | 6 | 1 | -1 | 6.9 | 1 | 60 | 90 | C |
| G177 | 1762,1764,1803 | 6 | 1 | 69 | 6.9 | 1 | 150 | 118 | C |
| G178 | 1753,1762,1764,1896 | 6 | 1 | 68 | 5.8 | 1 | 97 | 153 | C |
| G179 | 1762,1764 | 6 | 1 | 34 | 5.7 | 1 | 193 | 187 | C |
| G181 | 1762,1764 | 3 | 1 | 36 | 5.0 | 1 | 231 | 104 | C |
| G182 | 1764,1766,1803,1896 | 6 | 0 | 39 | 6.3 | 1 | 28 | 40 | C |
| G185 | 1753,1762,1764,1846,1896 | 6 | 1 | 47 | 5.3 | 0 | 28 | 44 | C |
| G186 | 1753,1762,1764 | 4 | 1 | -1 | -1.0 | 1 | 225 | 131 | C |
| G187 | 0 | 6 | 1 | 44 | 5.8 | 1 | 147 | 83 | B |
| G189 | 1762,1764 | 3 | 1 | 38 | 5.7 | 1 | 113 | 52 | C |
| G192 | 1753,1762,1764,1896 | 7 | 1 | 42 | 6.5 | 1 | 45 | 98 | C |
| G193 | 1764,1766,1896 | 6 | 0 | 59 | 6.1 | 1 | 160 | 132 | C |
| G195 | 1753 | 3 | 0 | 16 | 7.3 | 1 | 92 | 51 | B |
| G197 | 1753 | 5 | 1 | 67 | 7.0 | 1 | 46 | 33 | B |
| G198 | 1762,1764 | 6 | 1 | 47 | 6.3 | 1 | 31 | 45 | C |
| G200 | 1776,1846 | 6 | 1 | 38 | 6.6 | 1 | 37 | 45 | C |
| G203 | 1762,1764,1776,1896 | 6 | 1 | 46 | 6.6 | 0 | 79 | 100 | C |
| G204 | 1764 | 6 | 0 | 55 | 7.7 | 1 | 204 | 159 | C |
| G205 | 1753,1762,1764,1776,1896 | 6 | 1 | 43 | -1.0 | -1 | 72 | 100 | C |
| G206 | 1764 | 5 | 1 | 36 | 5.1 | 0 | 171 | NA | C |
| G207 | 1762,1764 | 7 | 1 | 55 | 6.0 | 1 | 37 | 69 | C |
| G209 | 1762,1764 | 3 | 0 | 30 | 6.9 | 1 | 221 | 74 | C |
| G211 | 1753,1762,1764 | 3 | 0 | 31 | 7.7 | 1 | 161 | 94 | C |
| G212 | 1762,1764 | 6 | 1 | 66 | 7.0 | 1 | 40 | 65 | C |
| G213 | 1762,1764 | 3 | 1 | 39 | 8.4 | 1 | 158 | 49 | C |
| G214 | 0 | 1 | 0 | 32 | 6.6 | -1 | 25 | 22 | B |
| G216 | 1762,1764 | 5 | 1 | 77 | 6.2 | 1 | 57 | 67 | C |
| G217 | 0 | 3 | 1 | 32 | 7.5 | 1 | 483 | 217 | C |
| G218 | 1762,1764,1896 | 6 | 1 | 46 | 6.8 | 0 | 94 | 116 | C |
| G219 | 1896 | 3 | 0 | 33 | 8.2 | 1 | 676 | 400 | B |
| G220 | 1753,1762,1764 | 6 | 0 | 46 | 6.6 | 1 | 152 | 110 | C |
| G221 | 1762,1764 | 4 | 1 | 38 | 7.5 | 1 | 330 | 98 | C |
| G222 | 1762,1803,1846 | 3 | 1 | 23 | 8.5 | 1 | 226 | 73 | C |
| G224 | 1762,1846,1896 | 4 | 1 | 29 | 6.3 | 1 | 19 | 30 | B |
| G225 | 1762,1764 | 6 | 0 | 54 | 7.4 | 1 | 61 | 105 | C |
| G228 | 1762,1764 | 6 | 0 | 60 | 6.8 | 1 | 43 | 78 | B |
| G232 | 1753,1762,1764,1803,1846,1899 | 6 | 1 | 48 | -1.0 | 1 | 39 | 51 | C |
| G233 | 1764,1766,1846 | 3 | 0 | 38 | 7.7 | 1 | 196 | 150 | C |
| G235 | 1762,1764,1896 | 3 | 1 | 32 | 6.4 | 1 | 94 | 44 | B |
| G237 | 1800 | 3 | 1 | -1 | 6.4 | 1 | 326 | 124 | B |
| G238 | 0 | 3 | 0 | 28 | -1.0 | 1 | 107 | 65 | B |
| G239 | 0 | 1 | 0 | 20 | 6.8 | 1 | 22 | 20 | B |
| G240 | 1762,1764,1899 | 5 | 1 | 46 | 5.9 | 0 | 27 | 38 | B |
| G242 | 1762,1766 | 7 | 1 | 41 | 6.3 | 0 | 22 | 59 | C |
| G243 | 0 | 3 | 0 | 33 | 7.5 | 1 | 203 | 107 | C |
| G244 | 0 | 1 | 0 | 22 | 8.0 | 1 | 23 | 32 | B |
| G245 | 0 | 1 | 0 | 20 | 8.0 | 1 | 26 | 29 | C |
| G246 | 0 | 1 | 0 | 20 | 7.7 | 1 | 11 | 15 | C |
| G247 | 0 | 1 | 0 | 20 | 3.0 | 1 | 13 | 52 | B |
| G249 | 1846,1896 | 3 | 1 | -1 | 7.4 | 1 | 222 | 86 | C |
| G250 | 1762,1764 | 3 | 1 | 25 | 7.7 | 1 | 39 | 28 | C |
| G251 | 0 | 3 | 1 | 37 | 9.1 | 1 | 210 | 66 | B |
| G252 | 1762,1764 | 4 | 1 | 30 | 8.0 | 1 | 374 | 73 | C |
| G253 | 1762,1764,1776 | 3 | 1 | 45 | 8.1 | 1 | 1104 | 203 | C |
| S01 | 0 | 1 | 0 | 39 | 3.0 | 1 | 19 | 21 | C |
| S02 | 0 | 3 | 0 | 18 | 5.2 | 1 | 12 | 15 | C |
| S06 | 0 | 3 | 1 | 41 | 3.0 | 1 | 79 | 35 | C |
| S07 | 1762,1764 | 3 | 1 | 36 | 6.7 | 1 | 36 | 66 | C |
| S08 | 1753,1762,1764,1896 | 7 | 1 | 53 | 4.6 | 1 | 411 | 383 | C |
| S12 | 1762,1764 | 1 | 1 | 30 | 8.0 | -1 | 105 | 60 | C |
| S14 | 0 | 1 | 0 | 29 | 3.0 | 1 | 11 | 14 | C |
| S15 | 1762,1764 | 3 | 1 | 18 | 6.8 | 0 | 367 | 128 | C |
| S17 | 1753,1762,1764,1896 | 3 | 1 | 44 | 3.0 | 1 | 29.9 | 26.8 | C |
| S21 | 0 | 3 | 0 | 36 | 3.0 | 1 | 19.6 | 21.1 | C |
| S22 | 1762,1764 | 3 | 1 | 14 | 6.8 | 1 | 100 | 146 | C |
| S23 | 1727,1762,1764 | 3 | 1 | 36 | 8.0 | 1 | 499 | 214 | C |
| S24 | 0 | 1 | 1 | 18 | 5.3 | 1 | 13.5 | 33.5 | C |
| S25 | 0 | 1 | 0 | 44 | 7.7 | 1 | 19.89 | 26.3 | C |
| S27 | 0 | 3 | 0 | 49 | 3.0 | 1 | 12.1 | 19.5 | C |
| S29 | 1762,1764 | 3 | 1 | 32 | 3.0 | 1 | 36.79 | 64 | C |
| S31 | 1762,1764 | 4 | 0 | 40 | 8.2 | 1 | 88.7 | 127.1 | C |
| S32 | 0 | 1 | 1 | 30 | 8.5 | 1 | 31 | 48 | C |
| S35 | 1762,1764,1803 | 6 | 1 | 43 | 6.7 | 1 | 46 | 25 | C |
| S36 | 1762,1764,1776 | 3 | 1 | 59 | 5.7 | 1 | 31 | 35 | C |
| S39 | 0 | 1 | 1 | 38 | 5.6 | 0 | 21.2 | 58.6 | C |

Diganosis, 1-ASC, 3-chronic hepatits, 4-liver failure, 5-liver cirrhosis without decompensation symptoms, 6-liver cirrhosis with decompensation symptoms,7-hepatocellular carcinoma; Gender, 1-Male, 0-Female; HBeAg, 0-negative,1-positive,-1-missing value; ALT, U/L; AST, U/L; -1, missing value.

**Table S2. Mutation profile in nt 1725-1900 based on PCR.**

| ***nt*** | ***Reference**** | **A** | **T** | **G** | **C** | **mutations** | **MAF** |
| --- | --- | --- | --- | --- | --- | --- | --- |
| 1764 | ***G*** | 109 | 1 | 53 | 0 | 110 | 70% |
| 1762 | ***A*** | 58 | 104 | 0 | 1 | 105 | 67% |
| 1727 | ***A*** | 84 | 19 | 55 | 6 | 80 | 51% |
| 1896 | ***G*** | 43 | 0 | 121 | 0 | 43 | 27% |
| 1799 | ***C*** | 0 | 0 | 28 | 136 | 28 | 18% |
| 1730 | ***C*** | 1 | 0 | 23 | 140 | 24 | 15% |
| 1753 | ***T*** | 6 | 139 | 1 | 18 | 25 | 16% |
| 1726 | ***A*** | 146 | 0 | 0 | 18 | 18 | 11% |
| 1752 | ***A*** | 146 | 1 | 17 | 0 | 18 | 11% |
| 1846 | ***A*** | 146 | 18 | 0 | 0 | 18 | 11% |
| 1776 | ***G*** | 8 | 1 | 155 | 0 | 9 | 6% |
| 1803 | ***T*** | 5 | 155 | 3 | 1 | 9 | 6% |
| 1758 | ***T*** | 0 | 158 | 0 | 6 | 6 | 4% |
| 1766 | ***C*** | 0 | 5 | 1 | 158 | 6 | 4% |
| 1809 | ***G*** | 2 | 0 | 158 | 4 | 6 | 4% |
| 1850 | ***T*** | 1 | 158 | 0 | 5 | 6 | 4% |
| 1756 | ***G*** | 1 | 0 | 159 | 4 | 5 | 3% |
| 1768 | ***T*** | 5 | 159 | 0 | 0 | 5 | 3% |
| 1742 | ***G*** | 1 | 3 | 160 | 0 | 4 | 3% |
| 1786 | ***G*** | 1 | 2 | 161 | 0 | 3 | 2% |
| 1858 | ***T*** | 0 | 161 | 0 | 3 | 3 | 2% |
| 1862 | ***G*** | 4 | 0 | 160 | 0 | 4 | 3% |
| 1864 | ***T*** | 2 | 161 | 0 | 1 | 3 | 2% |
| 1899 | ***G*** | 4 | 0 | 160 | 0 | 4 | 3% |

MAF, minor allele frequency, *with a HBV sequence (M12906) served as reference

**Table S3. GeneBank accession numbers for HBV sequences** submitted in this study

| **Patient** | **GenBank** |
| --- | --- |
| 3 | FJ938726 |
| 8 | FJ938727 |
| 14 | FJ938728 |
| 16 | FJ938729 |
| 24 | FJ938730 |
| 33 | FJ938731 |
| 38 | FJ938732 |
| 50 | FJ938733 |
| 69 | FJ938734 |
| 77 | FJ938735 |
| 79 | FJ938736 |
| 81 | FJ938737 |
| 83 | FJ938738 |
| 84 | FJ938739 |
| 85 | FJ938740 |
| 88 | FJ938741 |
| 95 | FJ938742 |
| 125 | FJ938743 |
| 135 | FJ938744 |
| 143 | FJ938745 |
| 144 | FJ938746 |
| 145 | FJ938747 |
| 152 | FJ938748 |
| 160 | FJ938749 |
| 163 | FJ938750 |
| 172 | FJ938751 |
| 182 | FJ938752 |
| 204 | FJ938753 |
| 210 | FJ938754 |
| 213 | FJ938755 |
| 217 | FJ938756 |
| 218 | FJ938757 |
| 221 | FJ938758 |
| 226 | FJ938759 |
| 234 | FJ938760 |
| 239 | FJ938761 |
| 240 | FJ938762 |
| 255 | FJ938763 |
| 256 | FJ938764 |
| 259 | FJ938765 |
| 260 | FJ938766 |
| 261 | FJ938767 |
| 262 | FJ938768 |
| 272 | FJ938769 |
| 273 | FJ938770 |
| 277 | FJ938771 |
| 278 | FJ938772 |
| 295 | FJ938773 |
| 296 | FJ938774 |
| 297 | FJ938775 |
| 299 | FJ938776 |
| 306 | FJ938777 |
| 314 | FJ938778 |
| 321 | FJ938779 |
| 340 | FJ938780 |
| 342 | FJ938781 |
| 344 | FJ938782 |
| 345 | FJ938783 |
| 351 | FJ938784 |
| 352 | FJ938785 |
| 353 | FJ938786 |
| 354 | FJ938787 |
| 356 | FJ938788 |
| 360 | FJ938789 |
| 366 | FJ938790 |
| 367 | FJ938791 |
| 370 | FJ938792 |
| G005 | FJ938793 |
| G045 | FJ938794 |
| G057 | FJ938795 |
| G060 | FJ938796 |
| G061 | FJ938797 |
| G067 | FJ938798 |
| G069 | FJ938799 |
| G071 | FJ938800 |
| G072 | FJ938801 |
| G073 | FJ938802 |
| G075 | FJ938803 |
| G076 | FJ938804 |
| G077 | FJ938805 |
| G079 | FJ938806 |
| G174 | FJ938807 |
| G175 | FJ938808 |
| G176 | FJ938809 |
| G177 | FJ938810 |
| G178 | FJ938811 |
| G179 | FJ938812 |
| G181 | FJ938813 |
| G182 | FJ938814 |
| G185 | FJ938815 |
| G186 | FJ938816 |
| G187 | FJ938817 |
| G189 | FJ938818 |
| G192 | FJ938819 |
| G193 | FJ938820 |
| G195 | FJ938821 |
| G197 | FJ938822 |
| G198 | FJ938823 |
| G200 | FJ938824 |
| G203 | FJ938825 |
| G204 | FJ938826 |
| G205 | FJ938827 |
| G206 | FJ938828 |
| G207 | FJ938829 |
| G209 | FJ938830 |
| G211 | FJ938831 |
| G212 | FJ938832 |
| G213 | FJ938833 |
| G214 | FJ938834 |
| G216 | FJ938835 |
| G217 | FJ938836 |
| G218 | FJ938837 |
| G219 | FJ938838 |
| G220 | FJ938839 |
| G221 | FJ938840 |
| G222 | FJ938841 |
| G224 | FJ938842 |
| G225 | FJ938843 |
| G229 | FJ938844 |
| G232 | FJ938845 |
| G233 | FJ938846 |
| G235 | FJ938847 |
| G237 | FJ938848 |
| G238 | FJ938849 |
| G239 | FJ938850 |
| G240 | FJ938851 |
| G242 | FJ938852 |
| G243 | FJ938853 |
| G244 | FJ938854 |
| G245 | FJ938855 |
| G246 | FJ938856 |
| G247 | FJ938857 |
| G249 | FJ938858 |
| G250 | FJ938859 |
| G251 | FJ938860 |
| G252 | FJ938861 |
| G253 | FJ938862 |
| S01 | FJ938863 |
| S02 | FJ938864 |
| S03 | FJ938865 |
| S04 | FJ938866 |
| S06 | FJ938867 |
| S07 | FJ938868 |
| S08 | FJ938869 |
| S12 | FJ938870 |
| S13 | FJ938871 |
| S14 | FJ938872 |
| S15 | FJ938873 |
| S17 | FJ938874 |
| S21 | FJ938875 |
| S22 | FJ938876 |
| S23 | FJ938877 |
| S24 | FJ938878 |
| S25 | FJ938879 |
| S27 | FJ938880 |
| S29 | FJ938881 |
| S30 | FJ938882 |
| S31 | FJ938883 |
| S32 | FJ938884 |
| S35 | FJ938885 |
| S36 | FJ938886 |
| S39 | FJ938887 |
| S40 | FJ938888 |
| S41 | FJ938889 |
| HCC20-3.fas | FJ938890 |
| HCC2-1.fas | FJ938891 |
| HCC2-2.fas | FJ938892 |
| HCC2-3.fas | FJ938893 |
| HCC2-4.fas | FJ938894 |
| HCC2-5.fas | FJ938895 |
| HCC3-1.fas | FJ938896 |
| HCC3-2.fas | FJ938897 |
| HCC3-3.fas | FJ938898 |
| HCC4-1.fas | FJ938899 |
| HCC10-1.fas | FJ938900 |
| HCC18-4.fas | FJ938901 |
| HCC17-3.fas | FJ938902 |
| HCC18-5.fas | FJ938903 |
| HCC18-2.fas | FJ938904 |
| HCC9-3.fas | FJ938905 |
| HCC6-3.fas | FJ938906 |
| HCC6-4.fas | FJ938907 |
| HCC6-5.fas | FJ938908 |
| HCC7-1.fas | FJ938909 |
| HCC7-2.fas | FJ938910 |
| HCC13-1.fas | FJ938911 |
| HCC7-3.fas | FJ938912 |
| HCC4-2.fas | FJ938913 |
| HCC4-3.fas | FJ938914 |
| HCC6-1.fas | FJ938915 |
| HCC6-2.fas | FJ938916 |
| HCC20-2.fas | FJ938917 |
| HCC8-1.fas | FJ938918 |
| HCC8-2.fas | FJ938919 |
| HCC9-1.fas | FJ938920 |
| HCC9-2.fas | FJ938921 |
| HCC9-4.fas | FJ938922 |
| HCC11-1.fas | FJ938923 |
| HCC12-1.fas | FJ938924 |
| HCC12-2.fas | FJ938925 |
| HCC14-1.fas | FJ938926 |
| HCC14-2.fas | FJ938927 |
| HCC15-1.fas | FJ938928 |
| HCC15-2.fas | FJ938929 |
| HCC16-1.fas | FJ938930 |
| HCC17-1.fas | FJ938931 |
| HCC17-2.fas | FJ938932 |
| HCC18-3.fas | FJ938933 |
| HCC18-1.fas | FJ938934 |
| HCC19-1.fas | FJ938935 |
| HCC19-2.fas | FJ938936 |
| HCC20-1.fas | FJ938937 |
| LC1-1.fas | FJ938938 |
| LC1-2.fas | FJ938939 |
| LC1-3.fas | FJ938940 |
| LC1-4.fas | FJ938941 |
| LC1-5.fas | FJ938942 |
| LC1-6.fas | FJ938943 |
| LC1-7.fas | FJ938944 |
| LC3-1.fas | FJ938945 |
| LC3-2.fas | FJ938946 |
| LC3-3.fas | FJ938947 |
| LC4-1.fas | FJ938948 |
| LC5-1.fas | FJ938949 |
| LC5-4.fas | FJ938950 |
| LC5-5.fas | FJ938951 |
| LC5-6.fas | FJ938952 |
| LC5-7.fas | FJ938953 |
| LC5-3.fas | FJ938954 |
| LC6-1.fas | FJ938955 |
| LC6-2.fas | FJ938956 |
| LC7-5.fas | FJ938957 |
| LC7-1.fas | FJ938958 |
| LC7-2.fas | FJ938959 |
| LC7-3.fas | FJ938960 |
| LC7-4.fas | FJ938961 |
| LC8-1.fas | FJ938962 |
| LC8-8.fas | FJ938963 |
| LC8-2.fas | FJ938964 |
| LC8-3.fas | FJ938965 |
| LC8-4.fas | FJ938966 |
| LC8-5.fas | FJ938967 |
| LC8-6.fas | FJ938968 |
| LC8-7.fas | FJ938969 |
| LC9-1.fas | FJ938970 |
| LC9-2.fas | FJ938971 |
| LC9-3.fas | FJ938972 |
| LC9-4.fas | FJ938973 |
| LC9-5.fas | FJ938974 |
| LC9-6.fas | FJ938975 |
| LC9-7.fas | FJ938976 |
| LC10-1.fas | FJ938977 |
| LC10-2.fas | FJ938978 |
| LC10-3.fas | FJ938979 |
| LC10-4.fas | FJ938980 |
| LC10-5.fas | FJ938981 |
| LC11-1.fas | FJ938982 |
| LC11-6.fas | FJ938983 |
| LC11-2.fas | FJ938984 |
| LC11-5.fas | FJ938985 |
| LC11-4.fas | FJ938986 |
| LC11-3.fas | FJ938987 |
| LC12-1.fas | FJ938988 |
| LC12-2.fas | FJ938989 |
| LC12-3.fas | FJ938990 |
| LC12-5.fas | FJ938991 |
| LC13-1.fas | FJ938992 |
| LC13-2.fas | FJ938993 |
| LC13-3.fas | FJ938994 |
| LC13-4.fas | FJ938995 |
| LC13-5.fas | FJ938996 |
| LC13-6.fas | FJ938997 |
| LC13-7.fas | FJ938998 |
| LC13-8.fas | FJ938999 |
| LC13-9.fas | FJ939000 |
| LC14-1.fas | FJ939001 |
| LC14-3.fas | FJ939002 |
| LC14-4.fas | FJ939003 |
| LC14-2.fas | FJ939004 |
| LC15-1.fas | FJ939005 |
| LC15-4.fas | FJ939006 |
| LC15-3.fas | FJ939007 |
| LC16-1.fas | FJ939008 |
| LC16-3.fas | FJ939009 |
| LC16-2.fas | FJ939010 |
| LC16-4.fas | FJ939011 |
| LC16-5.fas | FJ939012 |
| LC16-6.fas | FJ939013 |
| LC16-7.fas | FJ939014 |
| LC16-8.fas | FJ939015 |
| LC17-1.fas | FJ939016 |
| LC17-2.fas | FJ939017 |
| LC17-3.fas | FJ939018 |
| LC17-4.fas | FJ939019 |
| LC17-5.fas | FJ939020 |
| LC17-6.fas | FJ939021 |
| LC18-1.fas | FJ939022 |
| LC18-5.fas | FJ939023 |
| LC18-3.fas | FJ939024 |
| LC18-7.fas | FJ939025 |
| LC18-2.fas | FJ939026 |
| LC18-6.fas | FJ939027 |
| LC18-4.fas | FJ939028 |
| LC18-8.fas | FJ939029 |
| LC19-1.fas | FJ939030 |
| LC19-2.fas | FJ939031 |
| LC19-3.fas | FJ939032 |
